# Short-Term Transitions in First-Semester Motivation: A Three-Wave Study of Undergraduates’ Expectancy-Value-Cost Profiles

**DOI:** 10.3390/bs16101945

**Published:** 2026-10-19

**Authors:** Yi Jiang, Linjia Zhang, Xinyi Shi

**Affiliations:** 1 Department of Educational Psychology, Faculty of Education, East China Normal University, Shanghai 200062, China; 2 Department of Educational and Counselling Psychology, Faculty of Education, McGill University, Montreal, QC H3A 1Y2, Canada

**Keywords:** latent transition analysis, perceived costs, self-efficacy, situated expectancy-value theory, task values

## Abstract

Grounded in situated expectancy-value theory and a person-centered approach, the present study employed a three-wave longitudinal design to examine short-term transitions in first-year undergraduates’ expectancy-value-cost profiles across the first semester. Participants were 232 Chinese first-year undergraduates recruited through course-based convenience sampling from an introductory psychology course. Self-efficacy, task values, and perceived costs were assessed at three time points across the semester. Latent profile and latent transition analyses identified three recurring profiles: *Average-all*, *Burdened*, and *Positively motivated*. Approximately 51% of students remained in the same profile across the semester. Meaningful transitions were also observed. Specifically, transitions toward less favorable profiles were more apparent from T1 to T2, whereas transitions toward more favorable profiles were more apparent from T2 to T3. Gender did not significantly predict profile transitions. These findings suggest that first-year students’ expectancy-value-cost profiles can remain moderately stable while also shifting over short periods within a course. The results highlight the value of repeated assessment for understanding when students experience motivational burden and when their motivational configurations become more favorable.

## 1. Introduction

The first semester of undergraduate study represents a critical transitional period during which students must adapt to new academic expectations, unfamiliar learning environments, and greater responsibility for managing their own learning (Bryde & Milburn, 1990; Wu & Corpus, 2023). During this transition, students’ confidence in handling academic demands, the value they attach to coursework, and the burden they perceive in academic engagement may shift over time (Benden & Lauermann, 2022; Putwain & Sander, 2016). Understanding how students’ motivation changes during this period is therefore important for identifying when and how universities can provide more effective support.

Situated Expectancy-Value Theory (SEVT) provides a useful framework for examining these motivational changes. According to SEVT, students’ academic choices, engagement, persistence, and achievement-related pathways are shaped by competence-related beliefs, subjective task values, and perceived costs (Eccles & Wigfield, 2020, 2023). These motivational beliefs are situated in specific academic, social, and cultural contexts, and may change as students encounter new learning experiences. In the first semester of university, students may revise their beliefs about whether they can succeed, whether a course is interesting or useful, and whether academic engagement is burdensome.

Recent person-centered studies grounded in SEVT further suggest that expectancy, value, and cost beliefs do not operate in isolation. Rather, students hold distinct combinations of these beliefs that form qualitatively different motivational profiles (Lee et al., 2022; Jiang & Zhang, 2023; Perez et al., 2019). Although recent studies have identified meaningful expectancy-value-cost profiles among university students (Lee et al., 2022; Shankar et al., 2025), little is known about how these profiles change over shorter periods within a semester, particularly during students’ initial adjustment to university learning. Longitudinal person-centered approaches are therefore particularly useful for examining whether motivational profiles remain stable or are reshaped as students encounter ongoing course demands, assessment requirements, and new expectations for self-regulated learning (Xie et al., 2022).

Although longitudinal profile research has advanced understanding of students’ motivation, several gaps remain. First, longitudinal profile studies have often emphasized competence beliefs and positive task values while giving less attention to perceived costs (e.g., Lazarides et al., 2019, 2020). This omission is theoretically consequential because perceived costs are closely associated with avoidance behaviors and maladaptive academic outcomes (Jiang et al., 2018, 2020; Perez et al., 2014). Without cost indicators, profile analyses may underestimate the extent to which adaptive and maladaptive motivational beliefs coexist within students. Second, existing longitudinal expectancy-value-cost profile studies have focused mainly on secondary school students (Dietrich & Lazarides, 2019; Vinni-Laakso et al., 2022; Widlund et al., 2024) or the transition from primary to secondary school (Raufelder et al., 2022). Much less is known about first-year undergraduates, who are adjusting to university study. Third, many longitudinal profile studies have relied on two-wave designs (e.g., Dietrich & Lazarides, 2019; Vinni-Laakso et al., 2022; Widlund et al., 2024). Although two-wave designs can identify stability and change between two occasions, they provide limited information about whether transition patterns differ across successive periods. A three-wave design allows for a more differentiated, though still descriptive, examination of short-term profile stability and transition patterns across different phases of the semester.

To address these gaps, the present study examined the stability and transitions of first-year undergraduates’ motivational profiles across three time points during their first semester in an introductory psychology course. Specifically, we examined how self-efficacy, task values, and perceived costs combined into distinct motivational profiles and how students moved between these profiles over time. We also examined whether gender was associated with profile transitions.

### 1.1. Situated Expectancy-Value Theory

SEVT is a widely used framework for explaining how students’ competence-related beliefs and task values shape their academic choices, engagement, and achievement (Eccles & Wigfield, 2020; Wigfield & Cambria, 2010). Within this framework, expectancies for success refer to individuals’ perceptions of their likely performance on future tasks (Eccles & Wigfield, 2002). Self-efficacy, which measures students’ confidence in their academic abilities, is both conceptually and empirically aligned with expectancies for success (Bong & Skaalvik, 2003; Eccles & Wigfield, 2020). SEVT also distinguishes several components of subjective task value, including intrinsic or interest value, utility value, attainment value, and perceived cost. Interest value refers to the enjoyment derived from a task; utility value refers to the perceived usefulness of a task for current or future goals; attainment value reflects the personal importance of doing well on a task; and perceived cost captures the negative aspects of task engagement. Whereas interest, utility, and attainment values reflect positively valenced reasons for engaging in a task, perceived cost reflects the burdens, losses, and negative experiences associated with engagement.

Perceived cost historically received less attention than positive task values, particularly before the development of more systematic multidimensional cost measures. However, it is increasingly recognized as essential for understanding why students may lower their ambitions, reduce persistence, or develop avoidance motivation (Conley, 2012; Jiang et al., 2018, 2020). Cost is also multidimensional. Prior research has distinguished effort cost (negative appraisals of the effort required to complete a task), opportunity cost (lost opportunities to engage in other valued activities), ego or psychological cost (threats to the self-image associated with possible failure), and emotional cost (negative affective experiences during task engagement; Eccles et al., 1983; Wigfield et al., 2017; Wigfield & Eccles, 2020). Recent studies further reveal that examining these distinct facets of cost provides a more comprehensive understanding of student motivation (Gaspard et al., 2020; Lee et al., 2022; Perez et al., 2019). Thus, focusing only on positive task values may provide an incomplete account of students’ motivational adjustment during university transition.

### 1.2. Expectancy-Value-Cost Profiles Through a Person-Centered Lens

Research on expectancy-value beliefs has traditionally relied on variable-centered approaches. Such approaches are useful for examining average associations among constructs, but they are less suited to capturing how motivational beliefs are organized within individuals. Person-centered approaches, such as latent profile analysis (LPA), provide a complementary perspective by identifying subgroups of students with distinct configurations of motivational beliefs. As highlighted by Wormington and Linnenbrink-Garcia (2017), LPA is particularly effective for studying multifaceted motivation because it captures how multiple motivational beliefs combine within students.

Empirical research has identified heterogeneous expectancy-value-cost profiles. Common patterns include profiles with moderate levels across all beliefs, profiles with high competence-related beliefs and task values combined with low perceived costs, and profiles with low competence-related beliefs and task values combined with high perceived costs (e.g., Perez et al., 2019; Lee et al., 2022). Other studies have identified mixed motivational profiles, such as low perceived costs combined with low competence beliefs and values (Jiang & Zhang, 2023), or profiles characterized by simultaneously high competence beliefs, high task values, and high perceived costs (Conley, 2012; Watt et al., 2019). These findings show that motivation is not simply a matter of being high or low on a single dimension. Rather, students differ in the configurations of confidence, value, and burden they bring to academic tasks.

Longitudinal person-centered approaches, such as latent transition analyses (LTA), make it possible to examine whether these configurations are stable or change over time. This is particularly important because motivational change may involve movement between qualitatively different configurations rather than simple increases or decreases in individual variables (Xie et al., 2022). Some longitudinal studies have focused primarily on competence-related beliefs and positive task values, with less attention to perceived cost. For example, Fryer and Ainley (2019) identified *Low*, *Mid*, and *High* motivational subgroups among college students based on self-efficacy, self-concept, interest, and utility value. These subgroups were relatively stable across two semesters, although some students transitioned between *Low* and *Mid* groups. Lazarides et al. (2019) identified a motivational profile during the transition from Grade 9 to Grade 10 that they labeled the *Highly confident*, *hardly interested* profile, characterized by high self-concept, interest, and utility value. This profile, however, was less stable compared to others.

More recent studies have incorporated perceived costs into profile analyses. Dietrich and Lazarides (2019) identified four distinct profiles among secondary school students: *High motivation*, *Balanced above-average motivation*, *Average motivation*, and *Low motivation* (characterized by high perceived costs). These profiles remained relatively stable between Grades 9 and 10. Vinni-Laakso et al. (2022) investigated the stability of motivational profiles from Grades 7 to 8, finding that the *Moderate motivation and cost* profile was the most stable. Notably, students in profiles such as *High motivation with low cost*, *Low motivation with high cost*, or *High motivation with high cost* often transitioned to the *Moderate motivation and cost* profile, reflecting both positive and negative shifts influenced by perceived cost.

Other studies have confirmed the general stability of motivational profiles, while noting meaningful transitions. For instance, Raufelder et al. (2022) studied students transitioning from primary to lower secondary school and identified four profiles: *Positively engaged*, *Struggling ambitious*, *Disengaged relaxed*, and *Disengaged strained*. While most profiles remained stable, the *Positively engaged* profile showed a greater tendency to shift toward disengaged profiles over time. In a more recent study, Widlund et al. (2024) identified four motivational profiles among ninth-grade students: *Positively ambitious*, *Struggling ambitious*, *Indifferent*, and *Maladaptive*. Some students in the *Positively ambitious* profile transitioned to *the Struggling ambitious* profile, while others from the *Struggling ambitious* profile shifted to *Indifferent* or moved from *Indifferent* to *Maladaptive*. Positive changes were also observed, with some students transitioning from *Struggling ambitious* to *Positively ambitious* and others moving from *Maladaptive* to *Indifferent*.

These studies provide important evidence that expectancy-value-cost profiles can be stable but also open to change. However, relatively little is known about such transitions among first-year undergraduates. The first semester of university may involve substantial motivational recalibration as students encounter new academic demands, learn how university courses are assessed, and adjust their expectations about success, value, and cost. Moreover, many studies have focused primarily on transitions between two time points, providing limited insight into whether patterns of stability and change differ across successive measurement intervals. Examining transitions across multiple waves can therefore offer a more detailed understanding of short-term motivational dynamics and reveal whether different patterns of profile stability and change emerge over time.

### 1.3. Gender Differences in Profile Transitions

Gender differences have long been central to SEVT, which was originally developed in part to explain gendered patterns in students’ achievement-related choices, especially in mathematics and science pathways (Eccles, 1984; Eccles & Wigfield, 2023). From this perspective, gender may be associated not only with mean-level differences in expectancy and value beliefs, but also with the ways in which students’ motivational profiles are maintained or changed over time. Examining gender in longitudinal profile transitions can therefore help clarify whether motivational adjustment during the first semester of university follows similar or different patterns for male and female students.

Existing evidence on gender differences in motivational profile transitions remains limited and mixed. Dietrich and Lazarides (2019) found that boys were more likely than girls to remain in a high motivation profile throughout the school year. In contrast, Widlund et al. (2024) reported no significant gender differences in transition probabilities between profiles. These inconsistent findings suggest that gender may play different roles depending on educational stage, academic context, and the motivational indicators included in the profile model.

### 1.4. The Present Study

Building on SEVT and prior person-centered research, the present study examined the stability and change of expectancy-value-cost profiles among Chinese first-year undergraduates at three time points during their first semester. Students were enrolled in an introductory psychology course, and all motivational beliefs were assessed in relation to this course. This course-specific focus allowed us to examine short-term motivational transitions within a concrete academic context.

The study addressed three research questions. First, what expectancy-value-cost profiles can be identified among first-year undergraduates at three time points during the first semester? We examined profiles based on self-efficacy, three task values, and four perceived costs. Given the exploratory nature of person-centered profile identification, we expected multiple motivational profiles to emerge but did not propose a specific number or structure of profiles.

Second, how stable are students’ motivational profiles across the three waves, and what transition patterns characterize changes in profile membership? Based on previous longitudinal person-centered research, we expected profile membership to show moderate stability while allowing meaningful transitions among some students. Because the three-wave design allowed us to examine two successive transition intervals, we also explored whether patterns of stability and change differed across these intervals.

Third, is gender associated with students’ longitudinal profile transitions? Because prior evidence is limited and mixed, this question was examined without a directional hypothesis. By addressing these questions, the present study aims to clarify how first-year undergraduates’ self-efficacy, task values, and perceived costs are configured and change over time during the first semester, a formative period in students’ adjustment to university learning.

## 2. Method

### 2.1. Participants and Procedure

Participants were recruited through course-based convenience sampling from a required introductory psychology course at a public university in Shanghai, China. The course was taken by first-year undergraduate students majoring in education and focused on major theories and research methods in developmental psychology. The course met once a week and included lectures, assigned readings, student-led presentations, classroom discussions, and instructional feedback. Student-led presentations were distributed throughout the semester, and each student completed one small-group presentation based on assigned readings. Final grades were based on attendance and class participation (10%), a student-led presentation and discussion (20%), a reflective writing assignment (20%), and a paper-and-pencil final examination (50%).

Using a three-wave longitudinal design, students completed online questionnaires independently at three points during their first semester. All questionnaires assessed self-efficacy, task values, and perceived costs in relation to the course. The first wave of data collection (T1) was conducted during the 7th week of the fall semester and included 209 students. The second wave (T2) was conducted six weeks later, during the 13th week, and included 206 students. The reflective writing assignment was completed after T2. The third wave (T3) was conducted during the 19th week and included 190 students, followed by the final examination. Across the three waves, 232 students provided data for at least one measurement occasion and were included in the analytic sample. The mean age of participants was 19.40 years (*SD* = 0.96), and 28.4% were male. The semester timeline of data collection and major course activities is presented in Figure S2, and detailed longitudinal participation patterns are provided in the Online Supplemental Materials. Comparisons between participants with complete and incomplete longitudinal data showed generally similar T1 motivational characteristics. Incomplete cases reported higher opportunity cost, *t* (207) = 4.15, *p* < 0.001, and emotional cost, *t* (207) = 2.48, *p* = 0.014, whereas no significant differences were observed for the remaining baseline motivational indicators (all *p*s ≥ 0.141). Gender distribution also did not differ significantly between the two groups, *χ*^2^ (1) = 0.63, *p* = 0.428, Cramér’s *V* = 0.05. Including all students who provided data at one or more waves allowed us to make full use of the available longitudinal information in the latent transition analyses (Zhang et al., 2023; Zuidema et al., 2023). The study was approved by the Institutional Review Board for human participants at the authors’ university.

### 2.2. Measures

All survey items were written in Chinese and were based on six-point Likert scales ranging from 1 (*completely disagree*) to 6 (*completely agree*). Scales originally developed in English were translated and back translated following the process recommended by Brislin (1970). All survey items are presented in the Supplementary Materials.

#### 2.2.1. Self-Efficacy

Six items measuring self-efficacy were adopted from Bong (2008). The reliability coefficients for the three measurement waves were *α*s = 0.92, 0.92, and 0.95, respectively.

#### 2.2.2. Task Values

Items measuring task value beliefs were adopted from (Jiang & Zhang, 2023). There were twelve items assessing three types of task value, with four items each for interest value (*α*s = 0.95, 0.96, and 0.96 for the three measurement waves, respectively), utility value (*α*s = 0.93, 0.97, and 0.94 for the three measurement waves, respectively), and attainment value (*α*s = 0.86, 0.92, and 0.93 for the three measurement waves, respectively).

#### 2.2.3. Perceived Costs

Twelve items measuring students’ perceptions of cost were adopted from (Jiang et al., 2018). These items covered four facets: effort cost (*α*s = 0.88, 0.91, and 0.88 for the three measurement waves, respectively), opportunity cost (*α*s = 0.96, 0.98, and 0.97 for the three measurement waves, respectively), ego cost (*α*s = 0.94, 0.93, and 0.95), and emotional cost (*α*s = 0.81, 0.90, and 0.94 for the three measurement waves, respectively).

#### 2.2.4. Covariate

Gender was self-reported by students, with males coded as 0 and females coded as 1, and was included as a covariate in the analyses.

### 2.3. Statistical Analysis

All analyses were conducted using Mplus 8.10. The model parameters were estimated using the Maximum Likelihood Estimator (MLR) with Full Information Maximum Likelihood (FIML) estimation to handle missing data (Enders, 2010). Confirmatory factor analyses (CFA) were initially conducted on all measures to verify the factor structure of the constructs and obtain factor scores for subsequent profile analyses. CFA-derived factor scores were used to incorporate information from the validated measurement models while maintaining a tractable level of complexity in the longitudinal profile analyses (Morin et al., 2016; Wang et al., 2022). Before the profile analysis, longitudinal CFAs were conducted to test measurement invariance across the three time points, ensuring that students interpreted self-efficacy, task values, and perceived costs consistently over time. We specified a series of nested models with increasing invariance constraints and examined changes in goodness-of-fit statistics. First, we fitted a configural invariance model, with identical loading patterns but no parameter constraints. We then tested for weak (invariant factor loadings) and strong (invariant factor loadings and item intercepts) measurement invariance across the three waves. Measurement invariance was evaluated primarily based on changes in CFI, with changes in RMSEA considered as supplementary evidence. A change in CFI of no more than 0.010 and a change in RMSEA of no more than 0.015 were considered indicative of acceptable invariance (Chen, 2007; Cheung & Rensvold, 2002).

Model building using LTA began with identifying the best-fitting latent profiles by conducting a series of LPA separately for each time point. Several statistical indicators were used to identify the optimal number of profiles, following recommendations by Nylund et al. (2007): Model Log-Likelihood (LL), Akaike Information Criterion (AIC), Bayesian Information Criterion (BIC), and Sample-Size Adjusted BIC (aBIC), with lower values indicating better fit. The Vuong-Lo-Mendell-Rubin Likelihood Ratio Test (VLMR) and Bootstrapped Likelihood Ratio Test (BLRT) were also used, where a significant *p* value indicates a better fit for the k-profile model compared to the k-1 model. Additionally, entropy values greater than 0.70 indicate accurate classification. Beyond statistical indicators, conceptual interpretability and the practical significance of profile sizes were also evaluated to ensure a parsimonious solution in which each profile contained a substantial proportion of the sample.

In the next phase, LTA was used to examine transitions in profile membership across the semester, based on the cross-sectional LPA solutions (Widlund et al., 2024). Before examining profile transitions, we assessed longitudinal profile similarity following Morin and Litalien (2017). The profile solutions retained at each measurement occasion were incorporated into a longitudinal LPA. We first estimated a configural similarity model in which the same number of profiles was specified at each wave, while profile-specific indicator means were freely estimated across waves. We then tested structural similarity by constraining the means of corresponding profiles to equality over time. Model comparisons were based on the AIC, BIC, and aBIC, with lower values indicating a more favorable balance between model fit and parsimony.

To assess the role of gender in predicting these transitions, the BCH-LTA approach proposed by Asparouhov and Muthén (2021) was employed. The BCH method follows a two-step procedure: first, the latent profile measurement model is estimated and BCH weights are computed, accounting for the standard errors of the latent profile variable. In the second step, these weights are incorporated into an auxiliary model to examine the predictive effect of gender on profile transitions.

## 3. Results

### 3.1. Preliminary Results and Measurement Invariance

Descriptive statistics, reliability estimates, and correlations for all measures at each time point are presented in Table 1. Overall, the pattern of correlations was consistent with SEVT assumptions. Self-efficacy and task values were positively associated with one another, whereas perceived costs showed negative associations with self-efficacy and positive task values. At the descriptive mean level, self-efficacy and all three value dimensions decreased from T1 to T2 and then increased at T3. The cost dimensions showed a more differentiated pattern. Effort cost decreased at T2 but increased to a level slightly above T1 by T3. Opportunity cost and ego cost were also higher at T3 than at T1, whereas emotional cost remained relatively stable across the three measurement occasions.

As shown in Table 2, longitudinal metric invariance was supported for all study constructs across the three waves. Scalar invariance was also supported for task values and costs. For self-efficacy, full scalar invariance was not supported, but partial scalar invariance was established after one item intercept constraint was relaxed. These results indicate that the study constructs were generally comparable across measurement occasions, while also suggesting limited intercept noninvariance for self-efficacy.

### 3.2. Motivational Profiles and Their Longitudinal Similarity

Using factor scores obtained from the initial CFAs, we estimated LPA models with two to seven profiles separately at each measurement wave. Across the three waves, the three-profile solution was retained based on statistical fit, classification accuracy, profile size, parsimony, and substantive interpretability (see Table 3). AIC, BIC, and aBIC generally decreased as additional profiles were extracted, whereas the likelihood-ratio tests provided mixed evidence. Therefore, model selection was guided not only by statistical indices but also by whether the profiles were substantively meaningful and comparable across waves.

The three-profile solution was the most differentiated solution that produced a common set of clearly interpretable motivational configurations across the three measurement occasions. Classification accuracy was also high at each wave, with entropy values of 0.92, 0.93, and 0.95 at T1, T2, and T3, respectively. In contrast, solutions with more profiles did not yield an additional configuration that was consistently reproduced across waves. The additional classes that emerged in larger solutions varied in their substantive meaning across measurement occasions and, in some cases, were very small. For example, the four-profile solution at T1 included one class with only three students (1.3%; see Figure S1). Taken together, the three-profile solution provided the most parsimonious and substantively comparable representation of the data, allowing profiles to be meaningfully tracked in the subsequent LTA.

Before examining profile transitions, we tested longitudinal profile similarity across the three measurement occasions. The configural similarity model yielded AIC = 10,779.18, BIC = 11,158.32, aBIC = 10,809.68, and entropy = 0.94. Constraining the means of corresponding profiles to equality across time increased the AIC and aBIC, although the BIC decreased (AIC = 10,897.20, BIC = 11,110.90, aBIC = 10,914.39, entropy = 0.93). Given this mixed pattern, we retained the configural similarity model, in which profile-specific indicator means were allowed to vary across time. This indicates that the three profiles were substantively comparable across T1, T2, and T3, although the exact indicator means differed over time.

**Figure 1:**
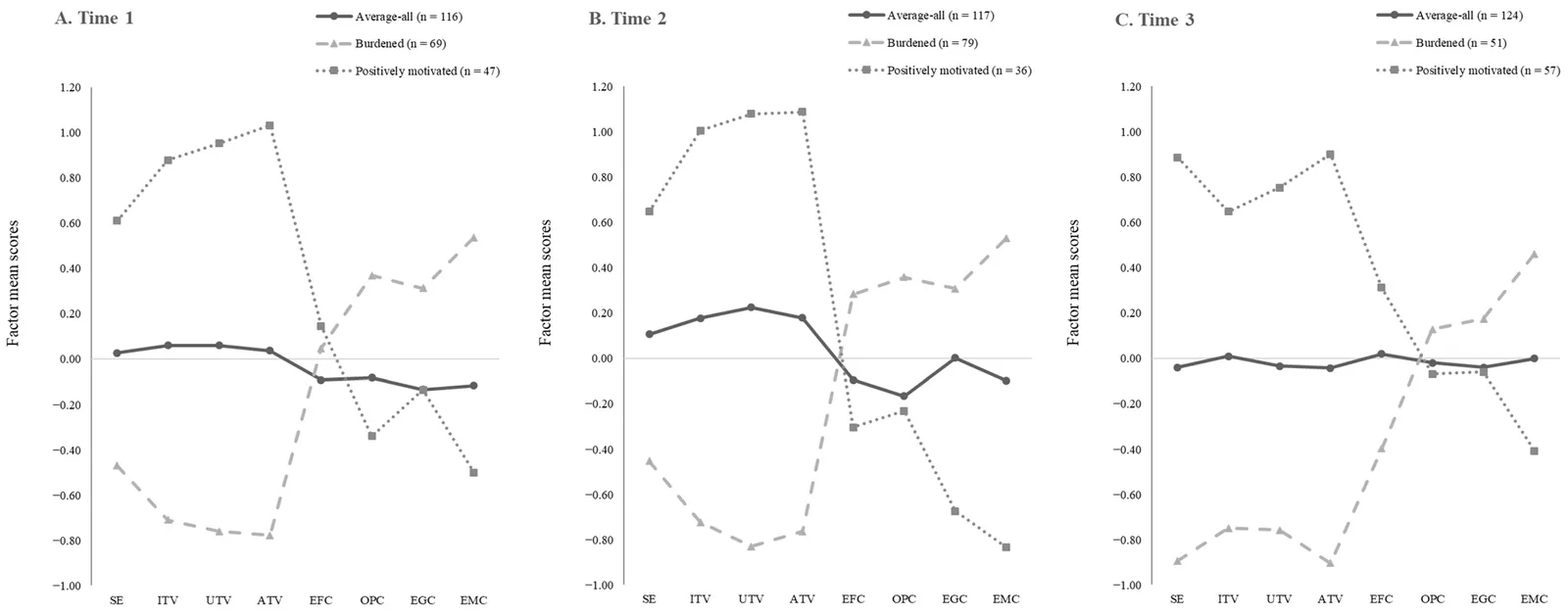
Motivational Profiles Across Three Measurement Waves. *Note.* SE = Self-efficacy; ITV = Interest value; UTV = Utility value; ATV = Attainment value; EFC = Effort cost; OPC = Opportunity cost; EGC = Ego cost; EMC = Emotional cost.

As shown in Figure 1, three qualitatively comparable motivational profiles emerged across the three measurement occasions: *Average-all*, *Burdened*, and *Positively motivated*. The *Average-all* profile showed levels of self-efficacy, task values, and perceived costs generally close to the sample average and represented the largest group at each wave (T1: 50.0%; T2: 50.4%; T3: 53.4%). The *Burdened* profile was characterized by relatively low self-efficacy and task values, together with relatively high perceived costs (T1: 29.7%; T2: 34.1%; T3: 22.0%). The *Positively motivated* profile was characterized by relatively high self-efficacy and task values, together with generally lower perceived costs (T1: 20.3%; T2: 15.5%; T3: 24.6%). Across waves, the profiles differed mainly in the magnitude of individual indicators rather than in their overall configurations.

### 3.3. Stability and Transitions in Profile Membership

Table 4 presents the transition probabilities between adjacent measurement occasions. Across both intervals, the largest transition probability for each profile was located on the diagonal, indicating substantial stability in profile membership. From T1 to T2, 77.7% of students in the *Burdened* profile and 73.0% of those in the *Average-all* profile remained in the same profile, whereas 56.3% of students in the *Positively motivated* profile remained in that profile. From T2 to T3, 72.8% of students in the *Average-all* profile and 72.4% of those in the *Positively motivated* profile remained in the same profile, compared with 51.8% of students in the *Burdened* profile. Thus, the *Average-all* profile showed relatively similar stability across the two intervals, whereas the numerical stability of the *Burdened* and *Positively motivated* profiles differed across the two observed periods.

When students changed profiles, transitions most often involved movement into the *Average-all* profile. From T1 to T2, 21.0% of students in the *Burdened* profile transitioned to the *Average-all* profile, whereas 20.3% of students in the *Average-all* profile moved to the *Burdened* profile and 6.6% moved to the *Positively motivated* profile. Among students initially in the *Positively motivated* profile, 41.6% transitioned to the *Average-all* profile. From T2 to T3, 38.0% of students in the *Burdened* profile transitioned to the *Average-all* profile, whereas 8.1% of students in the *Average-all* profile moved to the *Burdened* profile and 19.1% moved to the *Positively motivated* profile. Among students in the *Positively motivated* profile at T2, 27.6% transitioned to the *Average-all* profile at T3.

**Figure 2:**
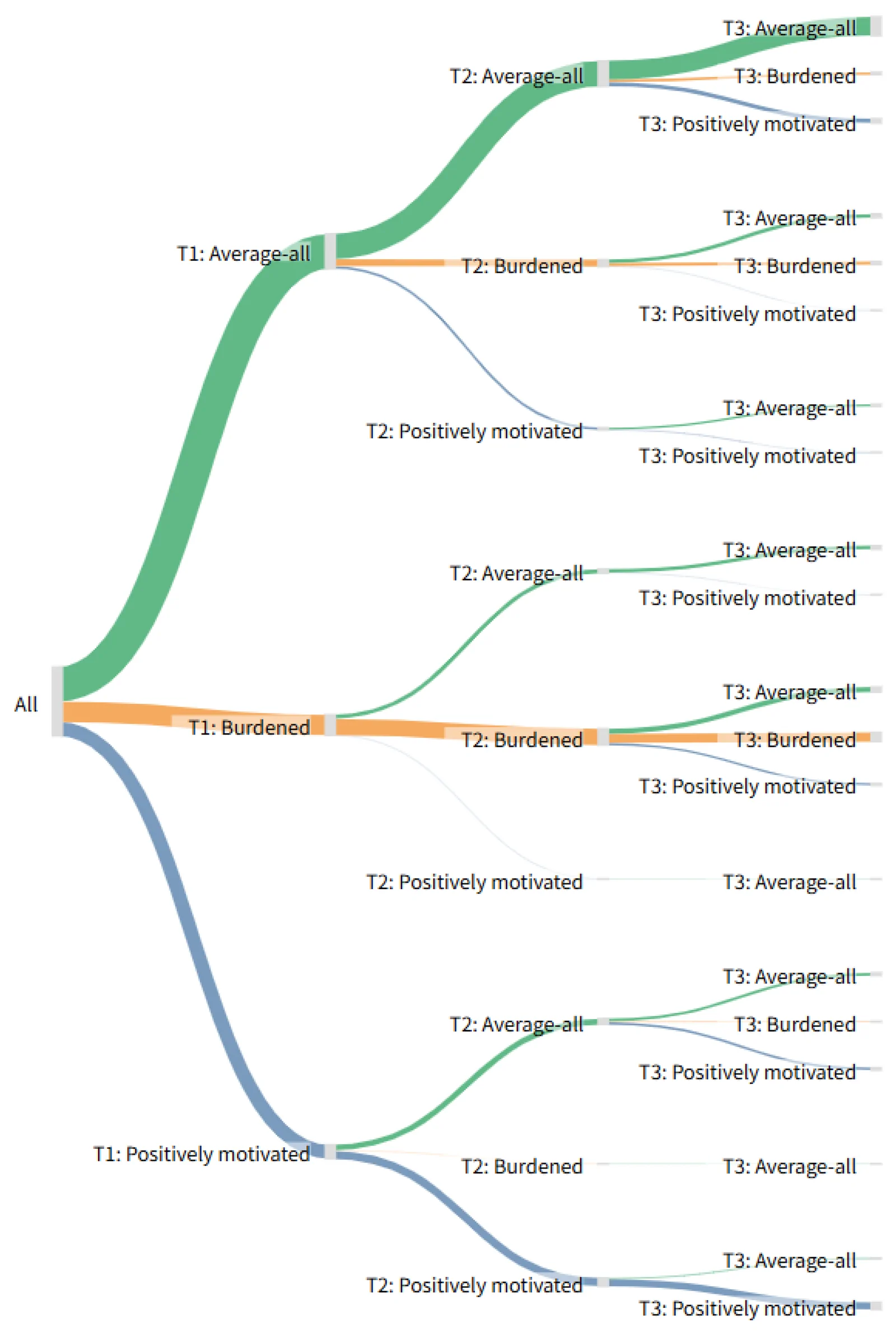
Shifts in Membership Transitions Across Three Time Points and Profiles. *Note.* The Sankey tree is read from left to right, beginning with the full sample (“All”) and proceeding through profile membership at T1, T2, and T3. Different colors distinguish the three latent profiles across measurement occasions. Each branch represents a subgroup of students following a particular profile pathway, with branch width proportional to subgroup size. No branch is shown for pathways with zero students.

To complement the model-estimated transition probabilities, Figure 2 presents complete three-wave pathways based on students’ most likely profile assignments using a Sankey tree (Sankey Tree, 2021). Table 4 shows transition probabilities between adjacent measurement occasions, whereas Figure 2 shows the number and proportion of students following each T1 to T2 to T3 profile sequence. These pathways should be interpreted as observed temporal sequences of profile membership, not as causal relations among profiles.

Profile proportions also changed descriptively across the semester. The *Burdened* profile increased from 29.7% at T1 to 34.1% at T2 and then decreased to 22.0% at T3. The *Positively motivated* profile showed the opposite numerical pattern, decreasing from 20.3% at T1 to 15.5% at T2 and then increasing to 24.6% at T3. The *Average-all* profile remained relatively similar in size across the three waves, accounting for 50.0%, 50.4%, and 53.4% of students at T1, T2, and T3, respectively. Taken together, the transition probabilities and profile proportions suggest a descriptive pattern in which less favorable motivational profiles were more prominent around the middle of the semester, whereas more favorable profiles were more represented toward the end of the semester.

### 3.4. Associations with Gender

As shown in Table 5, gender was not significantly associated with profile transitions from T1 to T2 or from T2 to T3. Across transition pathways, the confidence intervals for the odds ratios consistently included 1, indicating no statistically significant gender differences in transition probabilities.

## 4. Discussion

We adopted a longitudinal person-centered approach to examine first-year undergraduates’ expectancy-value-cost profiles across one semester in an introductory psychology course. Drawing on SEVT, we identified motivational profiles based on students’ self-efficacy, task values, and perceived costs. Three recurring and qualitatively comparable profiles emerged across the semester: *Average-all*, *Burdened*, and *Positively motivated*. Approximately half of the students remained in the same profile across all three time points, indicating moderate stability. At the same time, a substantial proportion of students moved between profiles, indicating that first-semester motivational configurations were open to short-term change. Descriptively, transitions toward less favorable profiles were more apparent from T1 to T2, whereas transitions toward more favorable profiles were more apparent from T2 to T3. Gender did not significantly predict profile transitions in the present sample.

### 4.1. Expectancy-Value-Cost Profiles Among First-Year Undergraduates

At the mean level, the motivational components showed differentiated patterns of change across the three waves. Self-efficacy and the three task value dimensions declined at the middle assessment and increased again at the final assessment. In contrast, perceived costs did not show the same pattern. Some cost dimensions remained stable or increased by T3, indicating that later increases in confidence and value did not necessarily coincide with a general reduction in motivational burden. A person-centered perspective complements these mean-level findings by showing how these components were configured within students over time. The LTA results identified three recurring and broadly comparable expectancy-value-cost profiles across the three waves, namely *Positively motivated*, *Average-all*, and *Burdened*. This three-profile structure is consistent with prior person-centered research showing that students’ motivational beliefs often organize into adaptive, moderate, and maladaptive configurations (Dietrich & Lazarides, 2019; Lee et al., 2022; Perez et al., 2019; Vinni-Laakso et al., 2022; Widlund et al., 2024). The *Positively motivated* profile, characterized by relatively high self-efficacy and task values together with low perceived costs, represents an adaptive motivational configuration. Students in this profile were more confident, placed greater value on the course, and perceived relatively little cost. In contrast, the *Burdened* profile was characterized by relatively low self-efficacy and task values together with higher perceived costs. This configuration reflects a more vulnerable motivational pattern in which students experienced lower confidence and value while perceiving greater effort, opportunity, ego, or emotional costs. The *Average-all* profile, which represented the largest group at all three waves, reflected moderate levels across expectancy, value, and cost beliefs.

The presence of a *Burdened* profile underscores the importance of including perceived cost in motivational profile research. If only self-efficacy and positive task values had been examined, students’ motivational difficulties might have been described simply as low confidence or low value. Including perceived cost shows that some first-year students experience course engagement not only as less valuable or less confidence-supportive, but also as effortful, costly, threatening, or emotionally difficult. This is consistent with SEVT research emphasizing that perceived cost is not merely the absence of value but a distinct component of motivation (e.g., Flake et al., 2015; Jiang & Zhang, 2023).

The *Average-all* profile was the largest at each wave, accounting for about half of the sample. This finding is consistent with previous studies identifying moderate or average motivational profiles among adolescents and undergraduates (Dietrich & Lazarides, 2019; Vinni-Laakso et al., 2022; Widlund et al., 2024). The prominence of this profile suggests that many Chinese first-year undergraduates do not show clearly adaptive or clearly maladaptive motivational configurations. Instead, they report moderate confidence, moderate task values, and moderate perceived costs. This group is educationally important because moderate motivation may appear unproblematic but still leave students vulnerable to movement toward more burdened profiles when academic demands increase.

### 4.2. Moderate Stability and Descriptive Variation in Profile Transitions

Profile membership showed moderate stability, with 50.9% of students remaining in the same profile across all three waves. This suggests that first-year undergraduates’ motivational profiles were neither fixed nor highly unstable. Many students maintained relatively consistent expectancy-value-cost configurations, whereas a substantial proportion moved between profiles. This finding aligns with prior longitudinal person-centered studies showing both stability and change in motivational profiles (Raufelder et al., 2022; Vinni-Laakso et al., 2022; Widlund et al., 2024; Xie et al., 2022).

This combination of stability and change is particularly relevant during the first semester of university, when students are adapting to new academic expectations, course structures, assessment practices, and demands for self-regulated learning. From a situated perspective on motivation, expectancy, value, and cost beliefs are responsive to students’ ongoing experiences within particular learning contexts (Hilpert & Marchand, 2018; Kaplan & Garner, 2020). The present findings extend this view by showing that short-term motivational change can be reflected not only in mean-level changes in individual constructs, but also in movement between broader configurations of expectancy, value, and cost beliefs.

The three-wave design also allowed us to describe how transition patterns differed across two successive intervals. From T1 to T2, transitions toward less favorable profiles were more apparent. The *Positively motivated* profile showed lower stability, with some students moving to the *Average-all* profile, and some students in the *Average-all* profile moved to the *Burdened* profile. From T2 to T3, transitions toward more favorable profiles were more apparent. Students in the *Burdened* profile more often moved to the *Average-all* profile, and students in the *Average-all* profile showed more movement toward the *Positively motivated* profile than toward the *Burdened* profile. These interval-specific patterns, however, should be interpreted descriptively, because the study did not formally test whether the T1 to T2 transition matrix differed from the T2 to T3 transition matrix.

These findings highlight the value of assessing students’ motivation at multiple points within a course rather than assuming that motivational profile membership remains stable throughout the semester. Movement both into and out of the *Burdened* profile suggests that students experiencing relatively high costs and lower self-efficacy or value do not necessarily represent a fixed group. At the same time, the observed shifts should be interpreted cautiously. The present study did not measure instructional support, feedback quality, workload, learning strategies, or intervention exposure. Future research could examine whether particular forms of course support, such as clarifying expectations, helping students manage workload, or providing timely and informative feedback, are associated with more favorable profile transitions at different points in the semester (Brinkworth et al., 2009; Lee et al., 2024).

### 4.3. Gender and Motivational Transitions

Gender did not significantly predict transitions among motivational profiles. In the present sample, male and female students appeared to show broadly similar patterns of motivational stability and change across the three waves. In this respect, the present results are consistent with Widlund et al. (2024), who also found no significant gender differences in profile transition probabilities.

This finding should nevertheless be interpreted cautiously. A non-significant gender effect does not demonstrate that gender differences are absent. Rather, it indicates that no significant gender differences in transition probabilities were detected in the present sample and course context. Gender differences in motivational transitions may also depend on the academic domain. Because the present study focused on an introductory psychology course, future research in more strongly gender-stereotyped domains, such as mathematics, engineering, or the physical sciences, could examine whether gender is more closely associated with profile transitions in those contexts.

The gender distribution of the present sample was also imbalanced, with more female students than male students. This imbalance may have limited statistical power to detect gender-related differences in transition probabilities. Future studies using larger and more gender-balanced samples across different academic domains are needed to examine whether gender is associated with motivational profile transitions in courses with different disciplinary meanings and evaluative demands.

### 4.4. Limitations and Future Directions

Several limitations should be acknowledged. First, the study was conducted with first-year undergraduates enrolled in a single introductory psychology course at one Chinese university. Although this context was well suited for examining short-term motivational profile transitions during the first semester, the relatively small and context-specific sample limits the generalizability of the findings. The focus on a single course also means that the observed profiles and transition patterns may partly reflect the demands, assessment structure, and disciplinary meaning of this particular learning context. Motivational profiles and transitions may differ across institutions, educational systems, and academic disciplines (Harackiewicz et al., 2008; Simpkins et al., 2006). Future research should examine whether similar patterns emerge in larger and more diverse samples and across courses with different instructional and evaluative features.

Second, although the three-wave design allowed us to examine short-term transition patterns within the semester, the study covered only one semester and began in Week 7. Therefore, the findings should not be interpreted as capturing students’ motivation from the very beginning of university study. Longer-term longitudinal research is needed to determine whether the observed changes reflect temporary adjustment processes within a course or more enduring motivational trajectories. Future studies with additional waves, including measurements before or at the very beginning of a course, could provide a more complete account of how students move toward or away from adaptive motivational profiles over time.

Third, several methodological limitations should be acknowledged. The use of CFA-derived factor scores as indicators in the LPA and LTA does not fully carry forward the uncertainty associated with factor score estimation into the subsequent mixture models, although factor scores incorporate information from the measurement models and provide some control for measurement error (Wang et al., 2022). Future research could examine the robustness of the present findings using fully latent factor mixture models, in which the measurement and profile components are estimated simultaneously. A further limitation concerns longitudinal profile similarity. Although the same three general motivational configurations were identified across measurement occasions, structural similarity was not retained. Corresponding profiles were therefore substantively comparable, but their exact indicator means differed over time. Transition probabilities should be interpreted with this distinction in mind, as reflecting movement between comparable rather than strictly identical motivational profiles.

Fourth, the present study examined gender as a covariate but did not include other individual or contextual predictors that may explain why students transitioned between profiles. Future research could investigate factors such as prior achievement, academic workload, perceived teacher support, peer support, autonomy support, and feedback experiences. Such work would help clarify the mechanisms underlying profile stability and change and inform more targeted interventions.

## 5. Conclusions

The present study examined stability and change in first-year undergraduates’ expectancy-value-cost profiles during the first semester. Across three waves in an introductory psychology course, approximately half of the students remained in the same profile, whereas others experienced at least one profile transition. Transition patterns differed descriptively across the two intervals. Shifts toward less favorable profiles were more apparent from T1 to T2, whereas shifts toward more favorable profiles were more apparent from T2 to T3. Gender did not significantly predict profile transitions in the present sample. Overall, these findings underscore the dynamic nature of students’ configurations of self-efficacy, task values, and perceived costs and highlight the value of examining motivational profiles at multiple points during a course.

## Figures and Tables

**Table 1:** Reliabilities, Descriptive Statistics, and Correlation Coefficients of Main Observed Variables.

	1	2	3	4	5	6	7	8	9	10	11	12	13	14	15	16	17	18	19	20	21	22	23	24
1 SE T1	--																							
2 ITV T1	0.47	--																						
3 UTV T1	0.48	0.74	--																					
4 ATV T1	0.58	0.73	0.79	--																				
5 EFC T1	0.10	0.02	0.05	0.06	--																			
6 OPC T1	−0.09	−0.27	−0.25	−0.20	0.46	--																		
7 EGC T1	−0.04	−0.11	−0.15	−0.11	0.33	0.45	--																	
8 EMC T1	−0.27	−0.37	−0.38	−0.34	0.43	0.63	0.69	--																
9 SE T2	0.47	0.31	0.31	0.38	−0.01	−0.12	−0.16	−0.32	--															
10 ITV T2	0.39	0.61	0.52	0.53	−0.06	−0.30	−0.18	−0.40	0.61	--														
11 UTV T2	0.32	0.51	0.56	0.59	−0.02	−0.28	−0.13	−0.37	0.57	0.76	--													
12 ATV T2	0.39	0.45	0.45	0.54	0.09	−0.16	−0.05	−0.28	0.61	0.74	0.84	--												
13 EFC T2	−0.12	−0.18	−0.22	−0.22	0.39	0.31	0.26	0.36	−0.18	−0.19	−0.12	−0.03	--											
14 OPC T2	−0.08	−0.23	−0.24	−0.23	0.24	0.41	0.21	0.29	−0.13	−0.24	−0.20	−0.07	0.63	--										
15 EGC T2	−0.15	−0.19	−0.24	−0.20	0.10	0.30	0.39	0.42	−0.20	−0.31	−0.26	−0.18	0.49	0.66	--									
16 EMC T2	−0.21	−0.29	−0.25	−0.28	0.18	0.36	0.33	0.51	−0.31	−0.40	−0.38	−0.28	0.44	0.57	0.78	--								
17 SE T3	0.46	0.37	0.36	0.47	0.08	−0.14	−0.07	−0.19	0.39	0.41	0.30	0.36	−0.14	−0.13	−0.12	−0.18	--							
18 ITV T3	0.46	0.42	0.37	0.50	0.08	−0.07	−0.08	−0.17	0.30	0.49	0.33	0.36	−0.27	−0.21	−0.19	−0.27	0.72	--						
19 UTV T3	0.47	0.40	0.46	0.54	0.02	−0.19	−0.02	−0.20	0.34	0.46	0.47	0.47	−0.18	−0.14	−0.10	−0.20	0.76	0.72	--					
20 ATV T3	0.41	0.39	0.43	0.50	0.12	−0.10	−0.02	−0.12	0.32	0.42	0.40	0.42	−0.13	−0.14	−0.09	−0.16	0.86	0.69	0.84	--				
21 EFC T3	0.09	0.01	−0.02	0.03	0.42	0.37	0.16	0.31	0.06	0.09	0.05	0.13	0.27	0.19	0.14	0.14	0.26	0.10	0.17	0.29	--			
22 OPC T3	−0.04	−0.15	−0.18	−0.17	0.25	0.51	0.21	0.38	−0.03	−0.13	−0.18	−0.06	0.32	0.36	0.26	0.29	0.00	−0.13	−0.10	0.00	0.63	--		
23 EGC T3	−0.05	−0.09	−0.14	−0.12	0.17	0.29	0.37	0.43	−0.10	−0.12	−0.16	−0.10	0.26	0.20	0.39	0.37	0.00	−0.10	−0.07	0.00	0.38	0.56	--	
24 EMC T3	−0.17	−0.24	−0.28	−0.27	0.19	0.40	0.35	0.52	−0.23	−0.30	−0.31	−0.21	0.37	0.33	0.40	0.47	−0.17	−0.30	−0.24	−0.16	0.45	0.67	0.71	--
*α*	0.92	0.95	0.93	0.86	0.88	0.96	0.94	0.81	0.92	0.96	0.97	0.92	0.91	0.98	0.93	0.90	0.95	0.96	0.94	0.93	0.88	0.97	0.95	0.94
*M*	4.49	4.71	4.85	4.88	3.86	2.76	2.86	2.68	4.34	4.57	4.81	4.74	3.65	2.87	2.88	2.74	4.84	5.00	5.10	4.99	3.95	2.99	3.02	2.62
*SD*	0.72	0.88	0.78	0.69	0.89	1.01	1.02	0.86	0.77	0.91	0.81	0.77	1.03	1.17	1.12	1.05	0.76	0.76	0.69	0.72	1.09	1.24	1.16	1.17

*Note.* SE = Self-efficacy; ITV = Interest value; UTV = Utility value; ATV = Attainment value; EFC = Effort cost; OPC = Opportunity cost; EGC = Ego cost; EMC = Emotional cost. All correlation coefficients with an absolute value greater than 0.14 are statistically significant.

**Table 2:** Fit Indices for Longitudinal Factorial Invariance Models.

Measurement Models	RMSEA [90% CI]	CFI	TLI	SRMR	*χ* ^2^	*df*	ΔCFI	ΔRMSEA
Self-efficacy								
1 Configural invariance	0.069 [0.058, 0.080]	0.933	0.923	0.045	278.26	132		
2 Metric invariance	0.069 [0.058, 0.080]	0.928	0.922	0.076	300.67	142	0.005	0.000
3 Scalar invariance	0.076 [0.066, 0.086]	0.908	0.907	0.080	354.44	152	0.020	0.007
4 Partial scalar invariance	0.072 [0.061, 0.082]	0.918	0.917	0.071	331.27	151	0.010	0.003
Task values								
1 Configural invariance	0.056 [0.050, 0.062]	0.933	0.925	0.050	959.41	558		
2 Metric invariance	0.055 [0.049, 0.061]	0.933	0.926	0.061	981.66	576	0.000	0.001
3 Scalar invariance	0.057 [0.051, 0.062]	0.927	0.923	0.068	1034.16	594	0.006	0.002
Costs								
1 Configural invariance	0.063 [0.057, 0.069]	0.922	0.907	0.060	1012.67	528		
2 Metric invariance	0.062 [0.056, 0.067]	0.923	0.911	0.059	1023.22	544	0.001	0.001
3 Scalar invariance	0.063 [0.058, 0.069]	0.917	0.906	0.060	1079.91	560	0.006	0.001

*Note.* RMSEA = root mean square error of approximation; CFI = comparative fit index; TLI = Tucker–Lewis index; SRMR = standardized root mean square residual; *df* = degrees of freedom; CI = confidence interval. ΔCFI and ΔRMSEA indicate absolute changes in model fit relative to the preceding less constrained model. For self-efficacy, the partial scalar model was compared with the metric model, and partial scalar invariance was established by releasing the equality constraint on the intercept of SE2 at Time 1.

**Table 3:** The Model Fit Results of Latent Profile Analyses at Three Measurement Points.

# of Profiles	LL	#Par	AIC	BIC	aBIC	Entropy	VLMR	BLRT	N Smallest Class
*Time 1*									
2	−1869.15	25	3788.30	3874.47	3795.24	0.82	0.05	<0.001	112, 48.3%
**3**	**−1728.03**	**34**	**3524.07**	**3641.26**	**3533.49**	**0.92**	**0.26**	**<0.001**	**48, 20.7%**
4	−1643.88	43	3373.76	3521.97	3385.68	0.95	0.31	<0.001	3, 1.3%
5	−1570.58	52	3245.17	3424.40	3259.58	0.96	0.23	<0.001	3, 1.3%
6	−1508.17	61	3138.33	3348.58	3155.25	0.93	0.19	<0.001	2, 0.9%
7	−1457.99	70	3055.97	3297.24	3075.38	0.94	10.00	<0.001	3, 1.3%
*Time 2*									
2	−2033.99	25	4117.97	4204.14	4124.90	0.92	0.06	<0.001	80, 34.5%
**3**	**−1903.22**	**34**	**3874.44**	**3991.63**	**3883.86**	**0.93**	**0.09**	**<0.001**	**19, 8.2%**
4	−1788.47	43	3662.94	3811.15	3674.86	0.97	0.65	<0.001	16, 6.9%
5	−1662.73	52	3429.47	3608.70	3443.89	0.98	0.03	<0.001	1, 0.4%
6	−1604.78	61	3331.55	3541.80	3348.46	0.98	0.81	<0.001	2, 0.9%
7	−1550.47	70	3240.94	3482.21	3260.35	0.98	0.51	<0.001	1, 0.4%
*Time 3*									
2	−1993.38	25	4036.76	4122.93	4043.69	0.93	<0.001	<0.001	64, 27.6%
**3**	**−1741.11**	**34**	**3550.23**	**3667.41**	**3559.65**	**0.95**	**<0.001**	**<0.001**	**50, 21.6%**
4	−1650.43	43	3386.85	3535.06	3398.78	0.96	0.01	<0.001	18, 7.8%
5	−1564.06	52	3232.11	3411.34	3246.53	0.94	0.42	<0.001	17, 7.3%
6	−1510.43	61	3142.85	3353.10	3159.76	0.94	0.36	<0.001	17, 7.3%
7	−1468.40	70	3076.80	3318.07	3096.21	0.94	0.17	<0.001	17, 7.3%

*Note.* LL = model log likelihood; #par = number of free parameters; AIC = Akaike information criterion; BIC = Bayesian information criterion; aBIC = Sample-Size adjusted BIC; VLMR = Vuong-Lo-Mendell-Rubin likelihood ratio test; BLRT = Bootstrapped likelihood ratio test. The selected model values are bolded.

**Table 4:** Latent Transition Probabilities and Odds Across Measurement Occasions.

	**Average-All T2**		**Burdened T2**		**Positively Motivated T2**	
	**Probability**	**Odds [95% CI]**	**Probability**	**Odds [95% CI]**	**Probability**	**Odds [95% CI]**
Average-all T1	0.730	1.000 [1.000, 1.000]	0.203	0.278 [0.128, 0.603]	0.066	0.091 [0.035, 0.234]
Burdened T1	0.210	0.270 [0.117, 0.623]	0.777	1.000 [1.000, 1.000]	0.014	0.017 [0.002, 0.166]
Positively motivated T1	0.416	0.739 [0.395, 1.383]	0.021	0.037 [0.005, 0.277]	0.563	1.000 [1.000, 1.000]
			
	**Average-All T3**		**Burdened T3**		**Positively Motivated T3**	
	**Probability**	**Odds [95% CI]**	**Probability**	**Odds [95% CI]**	**Probability**	**Odds [95% CI]**
Average-all T2	0.728	1.000 [1.000, 1.000]	0.081	0.111 [0.047, 0.264]	0.191	0.263 [0.156, 0.442]
Burdened T2	0.380	0.733 [0.418, 1.284]	0.518	1.000 [1.000, 1.000]	0.102	0.197 [0.091, 0.427]
Positively motivated T2	0.276	0.382 [0.152, 0.959]	0.000	0.000 [0.000, 0.000]	0.724	1.000 [1.000, 1.000]

*Note.* Transition probabilities are presented as point estimates without standard errors or confidence intervals. For each origin profile, remaining in the same profile serves as the reference category (odds = 1.000). The reported odds represent the likelihood of transitioning to an alternative profile relative to remaining in the original profile, with 95% confidence intervals provided for the odds.

**Table 5:** Covariate Effects of Gender on Transition Probabilities with Odds Ratios and Confidence Intervals from T1 to T2 and T2 to T3.

	**Latent Profile at T2**		
	**Average-all**	**Burdened**	**Positively motivated**
Latent Profile at T1			
Average-all	1.000 [1.000, 1.000]	1.224 [0.537, 2.789]	0.703 [0.251, 1.965]
Burdened	0.817 [0.359, 1.862]	1.000 [1.000, 1.000]	0.574 [0.188, 1.751]
Positively motivated	1.423 [0.509, 3.981]	1.742 [0.571, 5.314]	1.000 [1.000, 1.000]
			
	**Latent Profile at T3**		
	**Average-all**	**Burdened**	**Positively motivated**
Latent Profile at T2			
Average-all	1.000 [1.000, 1.000]	0.707 [0.301, 1.658]	0.666 [0.322, 1.380]
Burdened	1.415 [0.603, 3.320]	1.000 [1.000, 1.000]	0.943 [0.346, 2.570]
Positively motivated	1.501 [0.725, 3.110]	1.061 [0.389, 2.892]	1.000 [1.000, 1.000]

## Data Availability

The data from this study are available from the corresponding author upon reasonable request.

## Supplementary Materials

The following supporting information can be downloaded at: https://www.mdpi.com/article/10.3390/bs16101945/s1, Table S1: Items of Scales Used in the Present Study; Table S2: Longitudinal Participation Patterns Across the Three Waves; Table S3: Mean Factor Scores for Four-Profile Solutions Across Three Time Points; Figure S1: Four-Profile Solutions Across the Three Measurement Waves; Figure S2. Semester Timeline of Data Collection and Course Activities.
