# Supplementary material for: Short-Term Transitions in First-Semester Motivation: A Three-Wave Study of Undergraduates’ Expectancy-Value-Cost Profiles
Source: Behav Sci (Basel). 2026 Oct 19;16(10):1945. doi: 10.3390/bs16101945 (PMC13701842; doi:10.3390/bs16101945)
Supplement: Supplementary file 1 [file behavsci-16-01945-s001.zip › behavsci-4572956-supplementary.pdf]

Supplemental Materials

Table of Contents

Table S1: Items of Scales Used in the Present Study

Table S2: Longitudinal Participation Patterns Across the Three Waves

Table S3. Mean Factor Scores for Four-Profile Solutions Across Three Time Points

Figure S1: Four-Profile Solutions Across the Three Measurement Waves

Figure S2. Semester Timeline of Data Collection and Course Activities

Table S1

Items of Scales Used in the Present Study

**Items**
**Self-efficacy**
1. I'm certain that I can understand what is taught in the introductory psychology course.
2. I expect to do very well in the introductory psychology course.
3. I am sure that I can do an excellent job on the problems and tasks assigned for the introductory psychology course.
4. I know that I will be able to learn the material for the introductory psychology course.
5. I have excellent study skills for the introductory psychology course.
6. I think I will receive a good grade in the introductory psychology course.
**Interest value**
1. I find introductory psychology interesting.
2. I enjoy studying introductory psychology.
3. What I learn in the introductory psychology is fun.
4. I like introductory psychology.
**Utility value**
1. What I am learning in the introductory psychology is relevant to my life.
2. I think I will be able to use what I learn in the introductory psychology in other contexts.
3. What I am learning in the introductory psychology could be useful in everyday life.
4. I think introductory psychology would be useful to me in the future.
**Attainment value**
1. It is important for me to learn introductory psychology.
2. I feel that, to me, being good at solving problems which involve introductory psychology or psychological reasoning is important.
3. It is important for me to get good grades in introductory psychology.
4. Understanding introductory psychology is very important to me.
**Effort cost**
1. Doing well in introductory psychology requires more effort than I want to put into it.
2. It requires too much effort for me to get a good grade in introductory psychology.
3. It takes too much effort for me to do well in introductory psychology.
**Opportunity cost**
1. I have to give up other activities that I like to do well in the introductory psychology.

2. I have to sacrifice a lot of free time to be good at introductory psychology.
3. To do well in introductory psychology requires that I give up other activities I enjoy.

Ego cost

1. Others would think worse of me if I failed to do well in the introductory psychology.
2. Others would think I am incompetent if I get low grades in introductory psychology.
3. Others would be disappointed in me if I performed poorly in introductory psychology.

Emotional cost

1. Studying introductory psychology scares me.
2. Studying introductory psychology makes me feel stressed.
3. Studying introductory psychology makes me annoyed.

Table S2

Longitudinal Participation Patterns Across the Three Waves

Participation pattern	*n*	%
T1, T2, and T3	160	69.0
T1 and T2 only	25	10.8
T1 and T3 only	14	6.0
T2 and T3 only	14	6.0
T1 only	10	4.3
T2 only	7	3.0
T3 only	2	0.9
Total	232	100.0

Note. T1, T2, and T3 indicates participation in all three waves. "Only" indicates participation in the specified wave combination and no others.

Table S3

Mean Factor Scores for Four-Profile Solutions Across Three Time Points

Time / Profile	SE	ITV	UTV	ATV	EFC	OPC	EGC	EMC
Time 1								
Profile 1 (*n* = 3)	-0.22	-2.27	-2.27	-2.04	-0.01	1.23	1.04	1.11
Profile 2 (*n* = 79)	-0.45	-0.58	-0.66	-0.68	0.02	0.22	0.23	0.43
Profile 3 (*n* = 102)	0.06	0.10	0.12	0.09	-0.08	-0.05	-0.13	-0.13
Profile 4 (*n* = 48)	0.61	0.88	0.96	1.04	0.14	-0.33	-0.14	-0.50
Time 2								
Profile 1 (*n* = 18)	0.16	0.10	0.32	0.47	1.26	1.98	1.78	1.82
Profile 2 (*n* = 76)	-0.43	-0.71	-0.85	-0.81	0.24	0.28	0.24	0.44
Profile 3 (*n* = 102)	0.07	0.17	0.19	0.13	-0.27	-0.46	-0.23	-0.35
Profile 4 (*n* = 36)	0.61	0.95	1.08	1.08	-0.34	-0.26	-0.75	-0.83
Time 3								
Profile 1 (*n* = 29)	0.33	0.11	0.13	0.36	1.10	1.66	1.52	1.68
Profile 2 (*n* = 52)	-0.88	-0.74	-0.74	-0.88	-0.38	0.16	0.19	0.50
Profile 3 (*n* = 102)	-0.07	0.01	-0.02	-0.08	-0.17	-0.36	-0.34	-0.36
Profile 4 (*n* = 49)	0.89	0.70	0.76	0.90	0.13	-0.39	-0.37	-0.76

Note. SE = Self-efficacy; ITV = Interest value; UTV = Utility value; ATV = Attainment value; EFC = Effort cost; OPC = Opportunity cost; EGC = Ego cost; EMC = Emotional cost.

Figure S1

Four-Profile Solutions Across the Three Measurement Waves

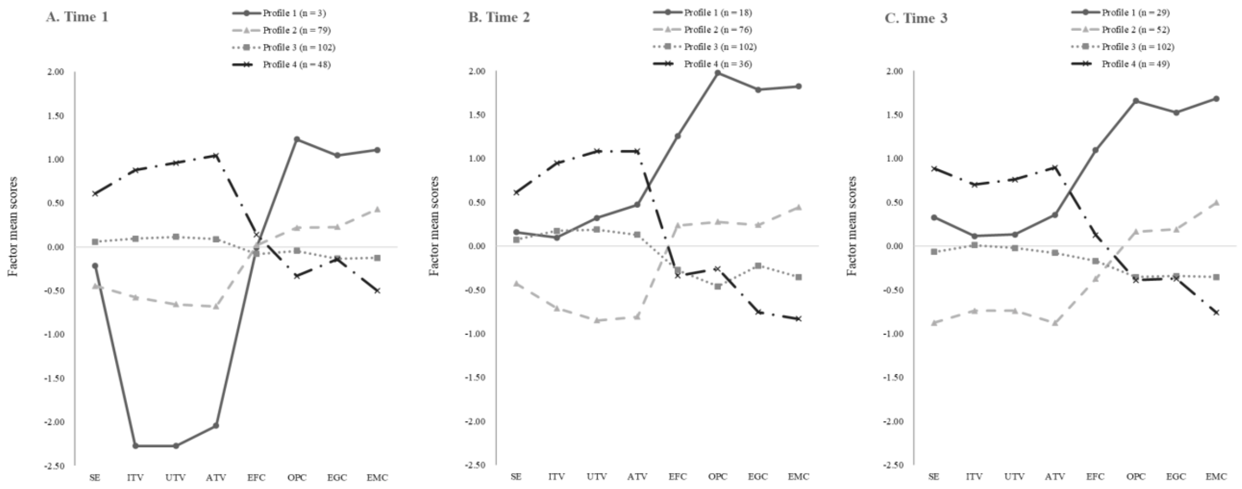

Note. SE = Self-efficacy; ITV = Interest value; UTV = Utility value; ATV = Attainment value; EFC = Effort cost; OPC = Opportunity cost; EGC = Ego cost; EMC = Emotional cost.

Figure S2

Semester Timeline of Data Collection and Course Activities

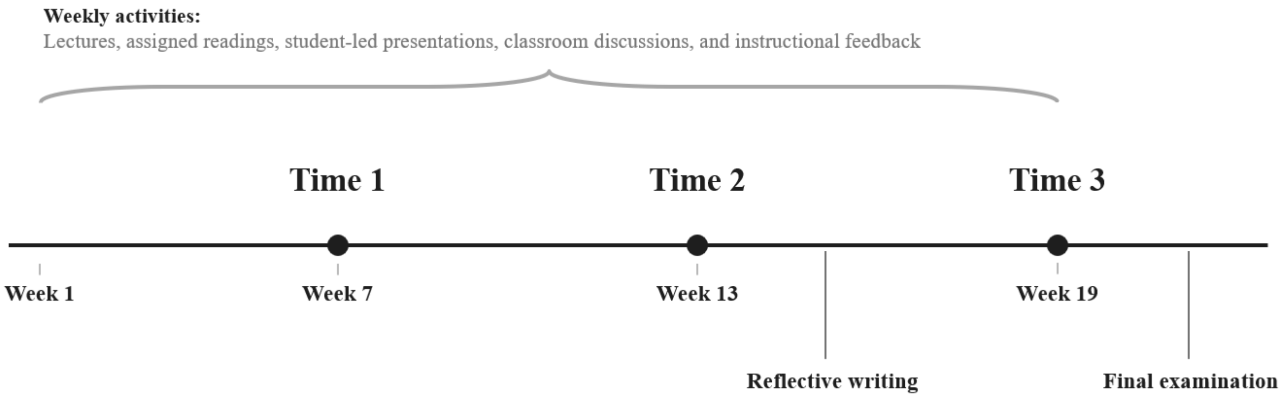

Note. Student-led presentations and instructional feedback were integrated into regular weekly class activities throughout the semester, with no designated major feedback sessions. Specific peaks in student workload were not systematically documented or assessed.
